# On the Vaccine Pock

**Published:** 1808-12

**Authors:** 


### *On the Vaccine Pock.*

THE first conjecture of Mr. Goodhall, on the origin of the cow-pock on the thigh of his little patient, (p. 334) must certainly have been correct, notwithstanding the statement of the mother. I believe the vaccine pock is never produced on the human subject but by actual application of the matter. Does the following little bit of my tediously lengthening out manuscript on vaccination, which you already announced in your Journal, and which still occupies my leisure hours, bear sufficiently on the present subject to be now inserted?

The vaccine pock is, of all the pimples, spots, or eruptions, which ever appear on the human body, the most distinctly characterised. It is *unique*. I know nothing that is imitative of it. We speak incorrectly in calling any other eruption spurious cow-pock. I am of opinion that all those which have been called secondary pocks, have arisen from an accidental secondary inoculation, as from a child's scratching itself at the inoculated part, and afterwards on some other part, with its nails thus charged with the vaccine ichor. An eminent and very early inoculator told me, that if a patient received a wound or scratch in any part of the body while under inoculation, a vaccine pock would arise at the part, from the system being generally affected. The notion was erroneous. An infant, a week after inoculation, caught a violent cold; the inflammation on the lungs was evident and most alarming. A blister was immediately applied to the chest. If the vaccine affection had been determined to the part so denuded, it must have killed the patient. The child was relieved, and recovered.

J. W.

*Salisbury Court; 5, x, 1808.*

*Remarkable*

